# Hirschsprung’s disease: Role of rectal suction biopsy - data on 216 specimens

**DOI:** 10.4103/0971-9261.70640

**Published:** 2010

**Authors:** Zillur Rahman, Jafrul Hannan, Saiful Islam

**Affiliations:** Department of Pathology, Chittagong Medical College, Chittagong, Bangladesh; 1 Department of Paediatric Surgery, Chittagong Maa-Shishu-O-General Hospital, Chittagong, Bangladesh

**Keywords:** Hirschsprung’s disease, suction biopsy, rectal biopsy

## Abstract

**Background:**

The diagnosis of Hirschsprung’s disease (HD) is dependent on the histological study of rectal ganglion cells, and an open rectal biopsy was the mainstay that required general anaesthesia (GA) and carried risk of postoperative rectal bleeding. Suction rectal biopsy later gained wide acceptance and became the choice as there is no requirement of GA and virtual absence of any complications.

**Materials and Methods:**

A retrospective review of the histological findings of 216 rectal suction biopsies studied from 2005 to 2009.

**Results:**

There were 143 male and 73 female children. 196 (90.7%) children were within 1 year of age. Among 216 rectal suction biopsies 181 (83.80%) were aganglionic, 27 (12.5%) were ganglionic and 8 (3.7%) were inadequate. Majority of patients were of less than 1 year of age (94.47%).

**Conclusions:**

The rectal suction biopsy is a bed side procedure, safe, cheap and time saving. There is high degree of accuracy, simplicity and absence of complications.

## INTRODUCTION

The definitive diagnosis of Hirschsprung’s disease (HD)is based on the findings of rectal biopsy characterizedby the absence of parasympathetic ganglia and the presence of hypertrophied nerve trunks in the bowelwall.[1–3] In recent years, suction biopsy has emerged asan important diagnostic tool.[4,5] This biopsy technique requires no general anaesthesia (GA) or suturing. So, many complications can be avoided. The procedure canbe conducted as an outpatient basis, is suitable for allages and is quite safe.[6]

Diagnostic accuracy depends on the adequacy of the specimen, level at which they were obtained, number of sections studied and the skill of the pathologist. When all the criteria are met, diagnostic accuracy reaches as high as 99.7%.[7] Hence suction biopsy becomes the initial and definitive modality used to evaluate the patient with constipation. The results with the use of this technique over the past five years are the subject of this study.

## MATERIALS AND METHODS

From January 2005 to December 2009, all specimens of rectal suction biopsy from children suspected to be having HD were studied. The specimens were received in 10% formalin solution and processed for paraffin embedding. Multiple serial sections were cut at 3–4 *µ*m thickness. About 8–10 sections per slide were mounted and two slides were made and were stained with H and E and then examined under light microscope.

On microscopic examination, biopsy specimens were classified as: (A) Ganglionic: clearly definable characteristic clusters of ganglion cells could easily be discerned in the sub-mucosa just beneath the mucosa after examination of 6–10 sections. (B) Aganglionic: when ganglion cells were absent. On an average 10 sections were examined before a comment being made as absence of ganglion cells. (C) Unsatisfactory or inadequate: biopsies were reported inadequate when: (i) No or very minimal sub-mucosa is present; (ii) Sub-mucosa is occupied by a lymphoid follicle; (iii) Specimen is decomposed; (iv) Specimen reveals stratified squamous epithelium (biopsy from too low position).

## RESULTS

A total 216 rectal suction biopsy specimens were studied, specifically for the evaluation of HD.

Analysis of rectal suction biopsies revealed 181 (83.80%) were aganglionic, 27 (12.5%) ganglionic and 8 (3.7%) specimens were inadequate for proper histopathological diagnosis. Age at the diagnosis ranged from 1 day to 3 years. Among them 196 (90.7%) were within 1 year of age. Age distribution in HD patients showed 60.22% within 1 month of age and 94.47% within 1 year of age [Table 1]. Among these 216 patients, 143 were males and 73 were females. Specimens diagnosed as HD were 181 with 122 male and 59 females (male female ratio is 2.08:1) [Table 2].

**Table 1:** Age group of patients and diagnosis (*n*=216)

Age group	HD (aganglionic)	HD excluded (ganglionic)	Inadequate specimen	Total
Up to 1 month	109	15	0	124 (57.41%)
1 month–1 year	62	07	3	72 (28.70%)
1 year–3 years	10	04	3	17 (07.87%)
>3 years	00	01	2	3 (01.39%)
Total	181 (83.8%)	27 (12.5%)	8 (3.7%)	216 (100.00%)

**Table 2:** Sex distribution of patients and diagnosis

Sex	HD (aganglionic)	HD excluded (ganglionic)	Inadequate specimen	Total
Male	122 (67.4%)	16	5	143
Female	59 (32.6%)	11	3	73
Total	181 (100%)	27	8	216

## DISCUSSION

Swenson[8,9] established rectal biopsy for the histological diagnosis of HD, which was supported in the followup studies with a 98% diagnostic accuracy.[10] Since then rectal biopsy was done frequently to diagnose HD definitively as well to differentiate HD from other causes of constipation. However, a full thickness rectal biopsy was needed to be done after a pre-operative preparation, GA and occasional hospitalization for rectal bleeding.

In 1960, Gherhardi[2] showed identical aganglionosis in the submucosal and myenteric plexuses. In 1968, Smith[11] demonstrated sequential maturation of myenteric plexus in a cranial caudal direction and pointed the importance of degree of maturation as well as presence or absence of ganglionic cells in the diagnosis of HD. Aldridge and Campbell in 1968[12] confirmed the presence of hypoganglionic zone within 1–2 cm of anal verge and demonstrated that the density of ganglion cells in the submucosal plexus was sufficient above this level. All these works contributed to develop an ideal suction rectal biopsy procedure.

In 1969, Noblett[5] first established suction rectal biopsy with a modified biopsy instrument. The instrument yields specimen 3.5–5 mm in diameter, with at least 2 mm of submucosa and was specifically suited for the diagnosis of HD. Later studies[13–17] established the accuracy of rectal suction biopsy. This study reemphasizes suction rectal biopsy for the definitive diagnosis of HD based on its 99.7% accuracy rate, simplicity of technique and absence of complications.

Age at the time of diagnosis of HD showed that 57.41% were below 1 month of age and 86.11% were below 1 years of age. Those rectal suction biopsies revealed aganglionic showed almost similar picture, 60.22% and 94.47% of patients were below 1 month of age and below 1 year of age, respectively. Ikeda and Goto in 1984[18] showed 48.7% of diagnosed cases were within 1 month of age and 60% were within 1 year of age.

About 181 cases (83.80%) in this study were diagnosed as HD by the absence of ganglion cells and/or presence of thickened nerve bundles in the submucosa. In 27 cases (12.50%) ganglion cells were demonstrated, so HD were excluded. 8 (3.7%) specimens were inadequate due to little or no submucosa. Hannan *et al*[17] Yunis *et al*[14] and Weintraub *et al*[15] reported inadequacy in 6.5, 15 and 10%, respectively in their studies. The rate of inadequate specimens in the present study is within justifiable range in comparison to the above studies.

Total eight inadequate samples were obtained. Of them 3 were from 1 month to 1 year, 3 were from 1–3 years and rest 2 were above 3 years of age. This result does not conform to the study of Croffie *et al*.[19] They found that the suction rectal biopsy is less likely to provide adequate submucosa for identification of ganglion cells after 3 years of age. But they worked only on children above 1 year of age. On the other hand, Pini-Pratoac *et al*[20] demonstrated that age does not represent a risk factor for inadequacy of the specimen.

## CONCLUSION

Rectal suction biopsy is a very simple, safe, quick, cheap and authentic technique for the diagnosis of HD. The procedure is quickly performed as outpatient basis in non-emergency situations. There is definitive diagnosis within 24 h and the hazards of GA can be avoided. High accuracy, simplicity and absence of complications strongly favour rectal suction biopsy as the procedure of choice for the diagnosis of HD.
